# Gatekeeping and referrals to cardiologists: General practitioners’ views on interactive communications

**DOI:** 10.3109/02813432.2013.784543

**Published:** 2013-06

**Authors:** Steinar Bjornsson, Johann A. Sigurdsson, Alma Eir Svavarsdottir, Gunnar Helgi Gudmundsson

**Affiliations:** ^1^Efstaleiti Primary Health Care Centre, Reykjavik, Iceland; ^2^Department of Family Medicine, University of Iceland and Centre of Development, Health Care Centre of the Capital Area Thönglabakka 1, Reykjavik, Iceland

**Keywords:** Gatekeeping, general practice, general practitioners, Iceland, opinions, primary health care, referral, rural medicine, views

## Abstract

**Objective:**

Referrals to specialists have not been compulsory in Iceland since 1984. In 2006, referrals were again required for patients to receive reimbursement for part of the cost of appointments with cardiologists. The aim of this study was to explore GPs’ attitudes to the referral system and possible professional gain by interactive communications.

**Design:**

Cross-sectional questionnaire survey.

**Setting, subjects, and main outcome measures:**

This is part of a larger study in 2007 on referrals from GPs to cardiologists. A questionnaire was sent to all working GPs in Iceland (n = 201 and responsible for 307 000 inhabitants) regarding the referral process, reasons for referrals, how often a response letter was received, and GPs’ attitudes to the referral system. Responses from doctors working in rural areas were compared with those working in Reykjavik and nearby urban areas.

**Results:**

The response rate was 63% (126 answers). The mean age of participants was 51; 89% were GP specialists and 60% worked in Reykjavik and nearby urban areas. Almost all respondents (98%) thought that report letters from cardiologists were helpful; 64% (95% confidence interval 53–73) thought that the recently introduced referral system did increase useful information that was beneficial to their patients. There was a statistically significant difference between colleagues working in rural areas and those working in Reykjavik and nearby urban areas regarding several aspects of the referral process.

**Conclusion:**

A referral system increases the flow of information and mutual communications between general practitioners and specialists to the benefit of the patients. The geographical location of the health care centre may be of importance regarding the value of the referrals.

The use of referral systems has been controversial. We lack knowledge on GPs’ professional evaluation of the usefulness of referrals in caring for their patients.

This study indicates that:

- Mutual communications between GPs and specialized physicians benefit patients.
- The geographical location of the primary health care centre may be of importance regarding the value of referrals.

## Introduction

Well-developed primary health care is an important link in comprehensive healthcare services [1–4]. There, general practitioners (GPs) play a key role in diagnosing and treating problems and serving as professional consultants to their clients about where to seek help for further health care, when pertinent. In such instances they refer their patients to specialized physicians for further diagnosis or treatment. GPs’ professional status is thought to be stronger where working in a referral system [5]. The referral systems have, however, usually been connected with the national health insurance system's participation in costs and are generally used to direct the flow of patients to specialists. Such a system exists in many countries [5–8]. The use of referral systems has been controversial in many parts of the world and both GPs and specialists differ in their views about such a system. There is still a lack of research on this matter, especially the pre-referral process as well as GPs’ professional evaluation of the usefulness of referrals in caring for their patients [5,9–15].

GPs in Iceland have not served as gatekeepers for nearly three decades as formal referrals to specialists have not been required since 1984. In 2006, however, referrals were required for patients to receive reimbursement for cardiologists’ fees. In our view, this primarily involved an administrative decision without a more detailed prelude, such as professional discussion among most physicians on the value of referrals as such. This gave a unique opportunity for further research into how decision-making of this kind is affected – for example, possible professional gains and the attitudes of patients, GPs, and specialists on the use of a referral system between GPs and other specialists. In the first part of this study we focused on our patients referred to cardiologists [10]. The response rate was 85% (209 out of 245). Eighty-nine per cent (95% CI = confidence interval 85–94) thought that the cardiologist should send their GP a letter concerning their visit. Our patients were used to a non-referral system before, and they were dissatisfied with the new reimbursement system for the cardiologist's fee. On the other hand, professional communications between GPs and cardiologists showed an eightfold increase in the form of referrals from GPs and a reply letter from the cardiologists, which was in accordance with the wishes of the patients [10].

The purpose of this study was to explore the attitudes of GPs in urban and rural areas towards the recently implemented system of referrals, and especially their professional reasons for the referrals to cardiologists and interactive gain.

## Material and methods

In 2007 a study was undertaken on referrals from GPs to cardiologists. In the first part of our study mentioned above we used the electronic medical record system from one health centre in Reykjavik to count referrals and the reply rate of the referrals from the cardiologists. This study was further completed with a survey sent to the patients [10]. This part, the second phase of our research, includes the processing of questionnaires sent to all practising GPs in Iceland in November 2007 (n = 201), which was about 1½ years after the initiation of the referral system. These GPs were considered to be responsible for primary care of the whole Icelandic population, amounting to 307 000 inhabitants at the time of the study. Sixty-three per cent (126/201) of all practising GPs in Iceland participated and 89% of them were specialists in general practice. There were missing data from 24 GPs regarding their workplace in rural versus urban areas, leaving analyses from 102 GPs for this part of our research, 62 (61%) from the urban area and 40 (39%) from the rural area.

The questionnaire contained 21 questions, including questions concerning gender, age, the physician's educational level, and the geographical location of the workplace. There were also questions about professional reasons regarding the (a) pre- referral process and the goal of their referrals (whether the physician was seeking medical assistance with diagnosis and treatment, or exclusion of a serious disease), as well as (b) possible professional gain, here defined as gain through referral feedback from the cardiologist, including better information regarding treatment options, or that it was helpful in general. Furthermore, some questions addressed the GPs’ views regarding possible gain through the change from the non-referral system to this new referral system. The responses of GPs in rural areas were compared with the responses of GPs in the urban area – here defined as Reykjavik, the capital city – and adjacent areas (including Reykjanesbaer).

Statistical calculations were done in Excel and SPSS. We used the level of 95% when calculating confidence interval for proportions. The chi-squared test or Fischer's exact test and Mann–Whitney U-test were used for statistical comparison of category variables. Significance was defined as p < 0.05.

The research was presented to the National Bioethics Committee, which ruled it to be outside its legal framework.

## Results

Table I shows the results of GPs’ opinion on the pre-referral and the goals of a referral system. They generally had the opinion that the initiative for their referral did not come from themselves, but rather from their patients or someone else. The majority of the GPs felt that they could have handled the matter themselves instead of making a referral (Table I). The attitudes of GPs in the urban area differed significantly from those physicians working in rural areas (Table I). Physicians in rural areas seldom lacked information about their patients when writing a professional referral to cardiologists, compared with physicians in the urban area (p < 0.05).

**Table I.** GPs’ opinions about the pre-referral process, and goals of a referral system to cardiologists: Those living in the urban area* are compared with those in the rural areas of Iceland (percentages; absolute numbers in parentheses).

	Total	Urban area*	Rural area	p-value
When I refer a patient, the situation is “rather often” or “very often” that the initiative comes from the patient, or the referral is not at my initiative	88 (110/125)	98 (61/62)	68 (27/40)	< 0.001
When I refer a patient, I “often” or “very often” feel that I could have handled the matter myself instead of making the referral	78 (93/120)	88 (51/58)	55 (22/40)	< 0.001
When I write a referral, it is “rather” or “very” seldom that the aim is to get specialist “help with diagnosis or treatment”, “to exclude serious disease”, or “to get better information about treatment options”	76 (91/120)	85 (51/60)	66 (25/38)	< 0.05
When the issue of referral arises, I “often” or “very often” lack information about the patient for the purpose of writing a referral of high quality.	30 (36/122)	41 (25/61)	18 (7/39)	< 0.05

Notes: *Reykjavik, Kopavogur, Hafnarfjördur, Gardabaer, Seltjarnarnes, Mosfellsbaer and Reykjanesbaer.

Possible professional gain from a referral system is shown in Table II. GPs were almost unanimous (98%; 95% CI 93–99) that information in response letters from cardiologists was helpful. In 63% of instances (95% CI 55–72) they also thought that the referral system increased the availability of useful medical information that was beneficial to patients. This opinion was stronger among GPs in the urban area compared with those of the rural GPs (Table II). Fifty per cent of GPs were satisfied with the change from no referral system to this type of referral system (Table II).

**Table II.** Possible professional gain from a referral system according to the GPs’ opinions: Those living in the urban area* are compared with those in the rural areas of Iceland (percentages; absolute numbers in parentheses).

	Total	Urban area*	Rural area	p-value
The new referral system is for the benefit of the patients as it adds to the GP's medical information	63 (77/122)	80 (48/61)	39 (15/38)	0.001
Information in the response letters from the cardiologists is helpful	98 (122/125)	97 (60/62)	98 (39/40)	NS
I am satisfied, “very much”, “rather much”, or “moderately” with the present referral system.	50 (61/123)	44 (27/62)	62 (24/39)	NS
GPs who estimate that they get a letter in more than 50% of cases from the cardiologists	64 (79/123)	54 (33/61)	82 (32/39)	0.01
I am more alert than before to the number of patients in my practice with cardiovascular diseases.	25 (31/124)	34 (21/62)	12 (5/41)	< 0.05

Notes: *Reykjavik, Kopavogur, Hafnarfjördur, Gardabaer, Seltjarnarnes, Mosfellsbaer and Reykjanesbaer. NS = not significant.

Ninety per cent of GPs would never, or very seldom, refuse to refer a patient. Physicians’ positions on the recently implemented referral system did not differ by gender or age (no significant difference).

## Discussion

GPs are apparently unanimous that mutual communications through referrals and specialists’ reply letters are beneficial in taking care of patients. GPs’ local conditions have a great deal to do with the pre-referral process. The findings indicate that in rural locations where there is less access to specialists (for example, greater distance and fewer specialists), rural GPs have more information about their patients with cardiovascular diseases and when they refer their patients to cardiologists they do it more often on their own initiative compared with their colleagues in the capital area. Professional communications in the form of referrals were possibly better in rural areas than in the urban area. A recent meta-analysis states that patients’ outcomes improved with good professional communications between GPs and specialists [16], and this is in harmony with our interpretation of the findings of this research.

The advantages of the referral system seem furthermore to be based on increased mutual communication between professionals. The disadvantages seem to be the reimbursement requirements incorporated in the new system in Iceland. It must be borne in mind that the implementation of the new referral system was an administrative decision without professional preparation or discussion within the medical profession. Demand for a referral letter probably leads to an increased workload if the initiative for the referral did not come from GPs themselves.

The response rate in this study is comparable to or better than that in various similar studies [14,15,17]. Although the number of participants seems small, it must be kept in mind that that the target group was all GPs in Iceland and their opinion is considered to have an effect on the whole Icelandic population.

The referral system introduced in 2006 has now been discontinued again. This is therefore the only research existing in Iceland on GPs’ views on a referral system from themselves to cardiologists. The strength of the research lies in this unique opportunity to investigate this change. Our research on referrals deals almost solely with the professional facets, i.e. the reasons for the referrals and the GPs’ attitudes. Our findings show that it is the conclusion of GPs as well as their patients [10] that mutual communications between general practitioners and specialists benefit patients. These findings are in agreement with comparable findings of time-tested and evidence-based medicine [16,18].
